# Predictors of non-exclusive breastfeeding at 6 months among rural mothers in east Ethiopia: a community-based analytical cross-sectional study

**DOI:** 10.1186/1746-4358-8-8

**Published:** 2013-08-07

**Authors:** Gudina Egata, Yemane Berhane, Alemayehu Worku

**Affiliations:** 1 College of Health and Medical Sciences, Haramaya University, Harar, Ethiopia; 2 Addis Continental Institute of Public Health, Addis Ababa, Ethiopia; 3 Department of Epidemiology and Biostatistics, School of Public Health, Addis Ababa University, Addis Continental Institute of Public Health, Addis Ababa, Ethiopia

**Keywords:** Non-exclusive breastfeeding, Ethiopia, Predictors, Children under-two

## Abstract

**Background:**

Exclusive breastfeeding in infants aged under six months is a simple and cost-effective feeding method that ensures better infant and child survival and boosts the achievement of child related Millennium Development Goals in the developing world. Identifying factors associated with good breastfeeding practice helps to increase its coverage and maximize its advantages through improved advocacy. The objective of this study was to identify the predictors of non-exclusive breastfeeding in the rural areas of eastern Ethiopia.

**Methods:**

A community-based analytical cross-sectional study was conducted on mother/caregiver–child pairs in east Ethiopia from July to August 2011. Data on infant feeding practices were collected by trained interviewers who used a pretested and structured questionnaire. Odds ratio with a 95% confidence interval was estimated for the predictors of non-exclusive breastfeeding using the multivariable logistic regression.

**Results:**

The prevalence of non-exclusive breastfeeding in infants aged under six months, was 28.3%. Non-exclusive breastfeeding was more likely to be practiced by mothers who were not married at the moment [AOR (95% CI) = 2.6 (1.1, 6.0)], mothers who had no access to health facility [AOR (95% CI) = 2.9 (1.9, 4.3)], and mothers whose knowledge about infant and young child feeding practices was low [AOR (95% CI) = 3.4 (2.4, 4.7)].

**Conclusion:**

Non–exclusive breastfeeding was more common among mothers with no marital relationships, poor access to health facilities, and inadequate knowledge about infant and young child feeding practices. Family support, education, and behavior change communication on infant feeding, especially on exclusive breastfeeding, at the community level may improve the knowledge, behavior, and practice of mothers on optimal infant and young child feeding practices.

## Background

Exclusive breastfeeding (EBF) in infants aged under six months is one of the optimal infant and young child feeding practices. According to the World Health Organization (WHO), EBF is feeding infants (0 –5 months of age) exclusively with breast milk (including milk expressed or from a wet nurse). Such children, WHO argues, may take only Oral Rehydration Salt (ORS), drops, and syrups (vitamins, minerals, medicines) in addition to their mothers’ milk [1]. EBF can save the lives of more than one million children under- five and improve the quality of their life [2-7]. It was found out that an estimated 1.3-1.45 million child deaths can be prevented annually through improved breastfeeding practices in low-income countries [6,8,9]. In addition to ensuring better infant and young child survival outcomes, this simple and cost-effective feeding method could help resource constrained settings achieve the child–health related Millennium Development Goals [3,10-12].

It is estimated that not more than 35% of infants worldwide are exclusively breastfed. The rate of exclusive breastfeeding which varies substantially worldwide [4,10,13,14] is very unsatisfactory in sub-Saharan Africa [5,6,10,15-20]. In Ethiopia, for instance, only half of the children are exclusively breastfed, with a median duration of 2.3 months and a mean duration of 4.2 months [12,21,22].

On the other hand, suboptimal breastfeeding (non-EBF) can decrease the full absorption of nutrients from breast milk and increase the risk of diarrhea and acute respiratory infections [23]. Non-EBF is also believed to be responsible for 10% of the disease burden, 10% of the 44 million Disability-Adjusted Life-Years (DALYs), and 1.4 million child deaths. Moreover, the long term impacts, such as poor academic performance, decreased productivity, and impaired cognitive and social development among children under-five years of age have been attributed to suboptimal Infant and Young Child Feeding Practices (IYCF) [24,25].

The causes of suboptimal breastfeeding are multitude and inter-related. In other studies, age, education, residence, marital status, occupation and smoking habit of the mother, and the household’s socio-economic position were identified as the distal predictors of non-exclusive breastfeeding [4,9,10,16,18,26-29]. On the other hand, maternal Antenatal Care (ANC) attendance, place of delivery, mode of delivery, knowledge about EBF, prelacteal feeding (feeding of infants with substances other than colostrum or first milk), timing of initiation of breastfeeding after delivery, colostrum feeding practice, caesarean delivery, smoking during pregnancy [4,9,10,12,15,16,18,19],[26-30], and the household food security status [31,32] were identified as maternal related risk factors. Moreover, sex, age, gestational age at birth, and birthweight of the child, diarrhea and Acute Respiratory tract Infection(ARI) were also found to be child related correlates of non–EBF in infants aged under six months [4,9,10,17-19,26,28,30].

Although documented evidence is scanty at all levels in Ethiopia, being single, unemployment, better socio-economic position, age of the child, and the practice of discarding colostrum were identified as the barriers to EBF [12,21,22,33,34]. Identifying factors associated with good breastfeeding practices in different contexts is assumed to facilitate better advocacy and wider coverage in the country. Therefore, this study was conducted to identify factors associated with non-exclusive breastfeeding in rural areas of eastern Ethiopia.

## Methods

The study was conducted at the Kersa Demographic Surveillance and Health Research Centre (KDS-HRC) of Haramaya University, east Ethiopia, from July to August 2011. In the Demographic Surveillance Site (DSS), 48192 adults and 7198 children aged under- five years lived in 10256 households during the study period.

The DSS which had three climatic zones, namely low land, midland and high land, was divided into two semi-urban and ten rural ‘kebeles’ (the smallest administrative units in Ethiopia). In Kersa district, there were no hospital and ambulance services, the nearest hospital being 50 km from the DSS. There were three health centers and ten community health posts within the geographic coverage of the DSS. Each community health post had two health extension or community health workers who provided basic primary health care services. The health care coverage of the district was 80% in 2010 [35,36].

Agriculture is the main means of livelihood for the study population. Crop production is basically on an annual basis, except in few locations where it is biannual. Sorghum and maize are the common grains cultivated, while potato and other vegetables are scarcely produced. Subsistent crops are often planted during the wet season (June-September) and harvested during the dry season (October–January). Khat, a stimulant plant with amphetamine like effects, is predominantly produced as a cash crop. Polygamous marital relationship are common with no profound cultural taboos related to eating habits.

A community-based analytical cross-sectional study was conducted on mothers of children under-two years of age (mother–child pair). The mothers and their children were initially selected at random from the DSS sampling frame. In addition to the random selection of the study participants, samples were proportionally allocated to the 12 ‘kebels’ of the DSS based on the total number of their households. If more than one under-five child lived in the selected household, one child was selected by a lottery method. The sample size was estimated using two approaches based on the objectives of the study. To estimate the prevalence of non-EBF in infants aged under six months a single population proportion formula was used with the following assumptions: expected prevalence of non-exclusive breastfeeding 51% [21,22], a 5% type I error, margin of error 5% (desired precision between sample and population parameter), and a 20% contingency for the non response. Accordingly, a sample size of 461 mother–child pair was calculated. On the other hand, to assess the predictors of non-exclusive breastfeeding, the sample size was estimated using Epi Info Version 3.5.1 with the following assumptions: a 5% type I error, power of the study 90%, control to case ratio 4:1 to detect the odds ratio of 2.0 among the cases which was estimated from the other study [22], being illiterate (main exposure) 66.1% among controls [37], and a 20% contingency for the non-response. Thus, the minimum sample size required for the study was 881 (220 mothers of non-exclusively breastfed children and 661 mothers of exclusively breastfed children). In this study, the latter sample size was considered to increase the power of the study. However, during the baseline survey for the longitudinal study, which was designed for a different study, the total number of mothers of children aged 6 to 23 months was found to be 860 (243 non-exclusively breastfed children and 617 exclusively breastfed children). To address other exposure variables and increase the power of the study, we included all the cases that were identified beyond the minimum requirement in the final analysis.

Data on socio-demographic and obstetric factors and infant feeding practices were collected using a structured and pre-tested interview questionnaire. The questionnaire was adapted from the Ethiopian Health and Demographic Survey (EDHS), WHO, and LINKAGE project questionnaires used to assess IYCF in developing countries. It was initially prepared in English and then translated into the local language, Afan Oromo, by fluent speakers of both languages, and it was translated back into English to check its consistency. A one week intensive training was given to 24 data collectors and 4 supervisors who were selected from KDS-HRC and the surrounding community. They were informed about the objective and relevance of the study, the confidentiality of the information, the respondent’s right, the questionnaire, the pre-test, the informed consent, and techniques to conduct the interview.

In this study, the outcome variable was the status of exclusive breastfeeding to infants under six months of age following the birth of the index child. EBF was understood as feeding only breast milk (including milk expressed or from a wet nurse) without anything else for the first six months of life with the exception of oral rehydration salt (ORS), drops, and syrups (vitamins, minerals, medicines) for therapeutic purposes [1]. Non-EBF was coded as “1” while EBF was coded as “0” for regression analysis.

The independent variables were age, education, residence, the marital status of the mother, the household socio-economic position and food security status, maternal access to a health facility, knowledge about IYCF, the practice of colostrum feeding, and the child’s sex.

The age of the mothers which was categorized into “15-34”and “35-49”years, was coded “0” and “1”, respectively. Illiterate mothers were coded “0” while the literates coded “1”. The “semi–urban” and “rural” residences of mothers were also coded “1” and “0”, respectively. Regarding their marital status, “others” was coded “0” and married “1”.

The household socio-economic position or wealth index was computed using 28 household related variables. They included the household assets, income, environmental health, water supply and dwelling. Principal component analysis (PCA) was performed to determine the households’ socio-economic position or wealth index. The categorical variables were made dummy before initiating the analysis, but the ordinal ones were ordered from the least important to the most important. Finally, the socio-economic position or wealth status was used in the consecutive analysis by classifying it into five groups: lowest, second (low), middle, fourth (high), and highest [12,38].

The household food security status was determined by computing nine standard household Food Insecurity Access Scale (HFIAS) questions adapted from the Food and Nutrition Technical Assistance (FANTA) Project of the United States Agency for International Development (USAID) which were designed for this purpose in 2007 [39]. All “Yes” responses were coded “1” and “No” responses were coded “0”, and the responses were summed up to obtain the household food insecurity index. The index which had a high internal consistency (Cronbach’s alpha = 0.90) [40], was further dichotomized as “food secure” and “food insecure” for a score equal to zero and greater than zero , respectively, and was coded “1” for “food secure” and “0” for “food insecure” for analysis [41].

The maternal access to health facilities was based on the mother’s and key informants’ estimate of the distance from the nearby health institution. A distance of 10 km radius was considered as access to health service facility and was coded “1” while a distance beyond this range of values was considered as lack of access and coded “0”.

The knowledge of mothers on IYCF was computed based on seven questions which included awareness of the mothers about the timing of breastfeeding initiation after delivery, exclusive breastfeeding, colostrum feeding and its importance, age at complementary feeding, how long breastfeeding should continue, and whether HIV sero-positive mother should breastfeed or not. The mothers who scored above the mean cut-off point were considered to have high knowledge about the practices and coded “1”, whereas those who scored below this cut–off point were considered to have low knowledge and coded “0” [29,42]. Likewise, last pregnancy was defined as a state of pregnancy for which a mother was pregnant for her index child. The mother’s colostrum feeding practice was categorized as “colostrum discarded” and coded “0”, and colostrum given to child was coded “1”.

The data were double entered using EpiData Version 3.1 by two data clerks and were exported to SPSS Version 16 for analysis. Descriptive statistics was used to summarize the study variables. Summary measures and proportions of the independent variables were computed against non-exclusive breastfeeding. The bivariate analysis was performed on important explanatory variables, and the crude odds ratio was calculated to identify the correlates of non-exclusive breastfeeding and to select candidate variables for the multivariable logistic regression analysis.

The Hosmer-Lemeshow goodness-of-fit and Omnibus tests of model coefficients tests with enter procedure were used to test for model fitness. The continuous variables, such as maternal and child age were tested for normal distribution using some statistical tests, including Kolmogorov-Smirnov and Shapiro-Wilk tests and through visual assessment, using the normal curve with a histogram. The explanatory variables were tested for multicollinearity before entering them into the multivariable model, using the Variance Inflation Factor (VIF) test, the Tolerance test, and values of the standard error.

Crude Odds Ratio (COR) with 95% confidence intervals was estimated to assess the association between each independent variable and the outcome variable, and a p-value was determined. Variables with p-value ≤ 0.2 in the bivariate analysis were considered in the multivariate analysis, along with variables that were well known predictors of non-exclusive breastfeeding, such as maternal age, and child’s sex, regardless of the cut-off point for p-value.

Adjusted Odds Ratio (AOR) with 95% confidence intervals was estimated to assess the strength of the association, and a p-value < 0.05 was used to declare the statistical significance in the multivariable analysis. Variables with p-value < 0.05 in the multivariable logistic regression analysis were considered as significant and independent predictors of non-exclusive breastfeeding.

The study was approved by the Ethical Review Committee of Haramaya University, College of Health and Medical Sciences, Ethiopia. The approval letter was dated 12 July, 2010, and numbered Ref IRERC/100/1/2010. The mothers of the children enrolled in the study were informed about the nature of the study, its objectives, expected outcomes, and benefits and the risks associated with it. Informed verbal and written consent was obtained from the mothers before the interview. Illiterate mothers consented by their thumb print after verbal consent. Privacy and confidentiality were maintained throughout the study.

## Results

A total of 860 mother-child pairs were included in the study, making a response rate of 97.7%. The prevalence of non-EBF in infants aged under six months was 28.3%. The mean (± SD) age of mothers of non-exclusively breastfed children was 27.1 (±5.6) years, while it was 26.7 (±5.6) years for mothers of exclusively breastfed children. The mean (± SD) age of non-exclusively breastfed children was 14.3 (±4.7) months while it was 13.6 (±4.5) months for exclusively breastfed children. The majority of the mothers (86.5%) were in the age group of 15-34 years while 70.8% of their children were in the age group of 12-23 months. A significant percentage of children who were non-exclusively breastfed had mothers with a rural background and lower educational levels (89.3% and 88.5% respectively) in contrast to their counterparts (Table 1).

**Table 1:** Socio-demographic and economic characteristics of the study participants by breast feeding Status, Kersa district, east Ethiopia, 2011

**Characteristics**	**Total**	**Breastfeeding status**	
		**Non –EBF (%)**	**EBF (%)**
Mother’s age ( years)			
15-34	744	208 (85.6)	536 (86.9)
35–49	116	35 (14.4)	81 (13.1)
Mean age (± standard deviation )	860	27.1 (5.6)	26.7 (5.6)
Mother’s residence			
Rural	745	217 (89.3)	528 (85.6)
Semi-urban	115	26 (10.7)	89 (14.4)
Mother’s education			
Illiterate	727	215 (88.5)	512 (83.0)
Literate	133	28 (11.5)	105 (17.0)
Marital status			
Currently unmarried ^¥^	27	13 (5.3)	14 (2.3)
Married	833	230 (94.7)	603 (97.7)
Mother’s occupation			
Farmers	538	150 (61.7)	388 (62.9)
Others^£^	322	93 (38.3)	229 (37.1)
Father’s education		123 (53.5)	319 (52.9)
Illiterate	442		
		107 (46.5)	284 (47.1)
literate	391		
Father’s occupation		220 (95.7)	568 (94.2)
Farmers	788		
		10 (4.3)	35 (5.8)
Others ^£^	45		
Household SEP			
Lowest	171	40 (16.5)	131 (21.2)
Second	173	57 (23.5)	116 (18.8)
Middle	172	37 (15.2)	135 (21.9)
Fourth	172	50 (20.6)	122 (19.8)
Highest	172	59 (24.3 )	113 (18.3)
Food security status			
Food insecure	397	93 (38.3)	304 (49.3)
Food secure	463	150 (61.7)	313 (50.7)
Sex of the child			
Male	447	124 (51.0)	323 (52.4)
Female	413	119 (49)	294 (47.6)
Age of the child ( in months)			
6-11	251	70 (28.8)	181 (29.3)
12-23	609	173 (71.2)	436 (70.7)
Mean age of the child (±SD)	860	14.3 (4.7)	13.6 (4.5)

^¥^ = separated, divorced, widowed, £ = civil servant, private employee, private business, daily laborer, student , *SEP* Socio-Economic Position.

The rate of non-exclusive breastfeeding was higher (34.2%) among children of mothers who had less access to health facility compared with the rate of exclusive breastfeeding. Non-exclusive breastfeeding was common (63.8%) among children of mother who had less knowledge about IYCF compared with exclusively breastfed children. Non-exclusive breastfeeding was also more common (11.9 %) among children of mothers who used to discard colostrum compared with exclusively breastfed children. Prelacteal feeding was practiced by 75.8% of the mothers (Table 2).

**Table 2:** Maternal characteristics by breastfeeding status, Kersa district, east Ethiopia, 2011

**Characteristics**	**Total**	**Breastfeeding status**	
		**Non-EBF**	**EBF**
		**n(%)**	**n(%)**
Mother’s access to a health facility			
No	161	83 (34.2)	78 (12.6)
Yes	699	160 (65.8)	539 (87.4)
ANC visit during last pregnancy			
No	492	108 (44.4)	384 (62.2)
Yes	368	135 (55.6)	233 (37.8)
Mother’s knowledge about IYCF			
Low	343	155 (63.8)	188 (30.5)
High	517	88 (36.2)	429 (69.5)
Colostrum feeding practice			
Discarded	73	29 (11.9)	44 (7.1)
Given to child	787	214 (88.1)	573 (92.9)
Prelacteal feeding			
Yes	652	167(68.7)	485(78.6)
No	208	76 (31.3)	132 (21.4)

*ANC* Antenatal care, *IYCF* Infant and Young Child Feeding.

The independent variables were evaluated for multicollinearity based on the results of VIF and tolerance tests and values of standard errors. Maternal ANC visit during last pregnancy tended to correlate with maternal access to health facility during the test for multicollinearity and excluded from the multivariable logistic regression model. However, after removing this variable from the model, the result of the VIF ranged from 1.017 to 1.211, while the values of the tolerance test fell to less than one, which was within the normal limit, showing the absence of multicollinearity [43]. Moreover, the values for the standard errors were small and less than two for all variables in the model, indicating the stability of the model.

The bivariate analysis revealed that maternal illiteracy [COR (95% CI) = 1.6 (1.0, 2.5)], being currently unmarried [COR (95% CI) = 2.4 (1.1, 5.3)], low (second) [COR (95% CI ) = 1.6 (1.001, 2.7)], and highest [COR (95% CI) = 1.7 (1.1, 2.8)] household socio-economic positions, household food security status [COR (95% CI ) =1.6 (1.2, 2.1)], less maternal access to health facility [COR (95% CI) = 3.6 (2.5, 5.1)], low maternal knowledge about IYCF practice [COR (95% CI) = 4.0 (2.9 , 5.5)], and discarding colostrum [COR (95% CI ) = 1.8 (1.08, 2.9)] were significantly associated with non-exclusive breastfeeding in infants aged under six months (p < 0.05) (Table 3).

**Table 3:** Predictors of non-exclusive breastfeeding among mothers of children under two years of age, Kersa district, east Ethiopia, 2011

**Characteristics**	**COR (95% CI)**	**AOR (95% CI)**
Mother’s age (years)		
15-34	1	1
35–49	1.1 (0.7, 1.7)	1.0 (0.6, 1.6)
Mother’s residence		
Rural	1.4 (0.9, 2.2)	1.4 (0.8, 2.4)
Semi-urban	1	1
Mother’s education		
Illiterate	1.6 (1.008, 2.5)*	1.5 (0.9, 2.5)
Literate	1	1
Marital status		
Currently unmarried	2.4 (1.1, 5.3)*	2.6 (1.1, 5.9)*
Married	1	1
Household SEP		
Lowest	1	1
Second	1.6 (1.001, 2.7)*	1.6 (0.9, 2.7)
Middle	0.9 (0.5, 1.5)	0.7 (0.4, 1.3)
Fourth	1.3 (0.8, 2.3)	0.9 (0.6, 1.6)
Highest	1.7 (1.07, 2.8)*	1.1 (0.6, 2.0)
Food security status		
Food insecure	1	1
Food secure	1.6 (1.2, 2.1)*	1.4 (0.9, 1.9)
Child’s sex		
Male	1	1
Female	1.1 (0.8, 1.4)	1.0 (0.7, 1.4)
Mother’s access to a health facility		
No	3.6 (2.5, 5.1)**	2.9 (1.9, 4.3) **
Yes	1	1
Mother’s knowledge on IYCF		
Low	4.0 (2.9, 5.5)**	3.4 (2.4, 4.7) **
High	1	1
Colostrum feeding practice		
Discarded	1.8 (1.1, 2.9)*	1.6 (0.9, 2.8)
Given to child	1	1

The odds of non-EBF in infants aged under six months were nearly 2 times higher among children of illiterate mothers and mothers who belonged to households in the second (low) and highest socio-economic positions. Similarly, the odds of non-EBF were 2.4 times higher among children of currently unmarried mothers compared with their counterparts. The odds of non-EBF were 1.6 times higher among children of mothers who belonged to food secured households compared with children of mothers who were from food insecure households. The odds of non-EBF were 4 times higher among children of mothers who had little access to health facilities and who had little knowledge about IYCF practices compared with their counterparts. Moreover, the odds of non-EBF were 1.8 times higher among children of mothers who used to discard colostrum than their counterparts (Table 3).

In the multivariable logistic regression model, after adjusting for all possible confounders, being currently unmarried [AOR (95% CI) =2.6, 95% CI (1.1, 5.9)], less maternal access to health facility [AOR (95% CI) = 2.9 ,95% CI (1.9 ,4.3)], and low maternal knowledge on IYCF practices [AOR (95% CI) = 3.4, 95% CI (2.4 ,4.7)] remained significant as independent predictors of non-exclusive breastfeeding in infants aged under six months (p < 0.05) (Table 3).

## Discussion

In this study, the prevalence of non-exclusive breastfeeding in infants aged under six months was 28.3%. We also examined the demographic and socio-economic characteristics, and the maternal and child related characteristics associated with the practice of non-exclusive breastfeeding for children under- two years in east Ethiopia. Accordingly, being currently unmarried, having less maternal access to health facility, and low maternal knowledge on IYCF practices were identified as independent predictors of non-exclusive breastfeeding.

Non-exclusive breastfeeding in infants aged under six months was practiced by 28.3% the mothers of children aged under-two years in this study. This finding was less than the findings of other previous studies conducted in developing countries ( in which the prevalence of non-EBF ranged from 28.7% to 88% [5,9,13,15,18,20,26,27],[30] including Ethiopia where it ranged from 51% to 75% [21,22,33]. This inconsistency could be due to the difference in the sample size and the age of the children included in the study as well as the retrospective nature of this study.

The marital status of mothers was associated with the practice of non-EBF in infants aged under six months. Children of currently unmarried mothers had nearly three times the odds of being not exclusively breastfed than their counterparts. This finding was in line with other similar studies in developing countries which identified that not living in joint families was a detriment to the practice of EBF [15,22,27]. This could be explained in such a way that unmarried mothers lack supportive advices to be offered on the importance of EBF by the husbands and other relatives compared with married mothers.

Non–EBF in infants aged under six months was associated with less maternal access to a health facility. Children of mothers who had little access to a health facility were found to be nearly 3 times more likely not to be exclusively breastfed compared with their counterparts. This might be explained by the fact that pregnant mothers could get information on EBF from health care providers during their contact for antenatal care services. This is possible only when mothers have adequate access to health facilities. In the principle of primary health care, accessibility refers to the availability of health service rendering institutions within 10 km radius. Nonetheless, maternal and child health service coverage was not enough in this study setting [10,44].

Low maternal knowledge on IYCF practices predicted the practice of non EBF in infants aged under six months. Children of mothers who scored below the mean cut-off point upon responding to IYCF knowledge questions were 3.4 times more likely not to be exclusively breastfed compared with their counterparts. No prior study described comprehensive maternal knowledge on IYCF practices, except one which identified low maternal knowledge on exclusive breastfeeding as the only predictor of non- exclusive breastfeeding [26,29,42].

In this study, maternal age and other maternal socio-economic characteristics, such as level of education, residence, and the household socio-economic position did not show significant association with non-EBF in infants aged under six months, unlike in other similar studies conducted earlier. This might be due to the fact that almost all the study participants in this study were homogenous with respect to their socio-economic characteristics. There was also no significant association between non-EBF and a child’s sex [4,9,12,15,24]. Moreover, non–EBF in infants aged under six months was not associated with household food security status. This finding was not in line with that of other similar studies in which household food security status has influenced infant and young child breastfeeding practices, particularly for infants and children greater than 6 months of life [31,32]. This variation could be attributed to the difference in methodological approach, in determining the food security status and the number of study participants considered in the respective studies.

One of the strengths of this study is its capacity to overcome the problem of selection bias; the non-exclusively breastfed children and the exclusively breastfed children were selected at random on the basis of KDS-HRC data base using a sampling frame. Its other strength is that the outcome variable of interest (EBF) was defined in the context of WHO’s IYCF indicators to select the appropriate subjects for the study. The baseline survey, for the purpose of other longitudinal study in the DSS, was also a good opportunity for the easy selection of the study subjects.

With regard to the limitations of the study, information on IYCF practices, including EBF, was collected retrospectively, which might have resulted in a recall bias as the majority of rural mothers might not evenly recall past events. This problem could have been avoided by using a prospective study design, in which mothers had been enrolled as soon as they had given birth and followed for their practice of EBF in infants aged under six months. However, as child birth is a special event, this limitation might have had only little effect on the findings of the study. Similarly, interviewer bias might have also occurred during the interviews. However, since due attention was given to the entire procedure of data collection, its effect might not be a threat to the findings of the study.

## Conclusion

The results of this study indicated that the prevalence of non-EBF, in infants aged under six months, was 28%. It was common among mothers with no marital relationship, poor access to health facilities, and inadequate knowledge about Infant and Young Child Feeding Practices. Family support, education, and Behavior Change Communication on infant feeding at the community level may improve the knowledge, behavior, and practice of the mothers on optimal Infant and Young Child Feeding Practices, particularly on exclusive breastfeeding.

## Competing interests

The authors declare that they have no competing interests.

## Authors’ contributions

GE: participated in the design of the study, performed the data collection, performed the statistical analysis and served as the lead author of the manuscript. YB: participated in the design of the study and contributed to finalization of the manuscript. AW: participated in the design of the study, statistical analysis and in finalizing the manuscript. All authors read and approved the final manuscript.
